# Mechanisms That Underly T Cell Immunity in Graves’ Orbitopathy

**DOI:** 10.3389/fendo.2021.648732

**Published:** 2021-04-01

**Authors:** Sijie Fang, Yi Lu, Yazhuo Huang, Huifang Zhou, Xianqun Fan

**Affiliations:** ^1^ Department of Ophthalmology, Shanghai Ninth People’s Hospital, Shanghai JiaoTong University School of Medicine, Shanghai, China; ^2^ Shanghai Key Laboratory of Orbital Diseases and Ocular Oncology, Shanghai Ninth People’s Hospital, Shanghai JiaoTong University School of Medicine, Shanghai, China; ^3^ Shanghai Institute of Immunology, Shanghai JiaoTong University School of Medicine, Shanghai, China; ^4^ Department of Immunology and Microbiology, Shanghai JiaoTong University School of Medicine, Shanghai, China

**Keywords:** Graves’ orbitopathy, thyroid-associated ophthalmology, T cell immunity, effector T cell, orbital fibroblast, fibrocyte

## Abstract

Graves’ orbitopathy (GO), also known as thyroid-associated ophthalmopathy, is the most common ocular abnormality of Graves’ disease. It is a disfiguring, invalidating, and potentially blinding orbital disease mediated by an interlocking and complicated immune network. Self-reactive T cells directly against thyroid-stimulating hormone receptor-bearing orbital fibroblasts contribute to autoimmune inflammation and tissue remodeling in GO orbital connective tissues. To date, T helper (Th) 1 (cytotoxic leaning) and Th2 (antibody leaning) cell subsets and an emerging role of Th17 (fibrotic leaning) cells have been implicated in GO pathogenesis. The potential feedback loops between orbital native residential CD34^-^ fibroblasts, CD34^+^ infiltrating fibrocytes, and effector T cells may affect the T cell subset bias and the skewed pattern of cytokine production in the orbit, thereby determining the outcomes of GO autoimmune reactions. Characterization of the T cell subsets that drive GO and the cytokines they express may significantly advance our understanding of orbital autoimmunity and the development of promising therapeutic strategies against pathological T cells.

## Introduction

Graves’ orbitopathy (GO), also known as thyroid-associated ophthalmopathy, is the ocular abnormality of Graves’ disease (GD). The prevalence of GO in Europe is about 10/10,000 people, which is above the threshold for rarity in Europe (1). However, as the most common extrathyroidal complication, GO affects 25-30% of patients with Graves’ hyperthyroidism and detailed orbital imaging has revealed orbital soft tissue changes in 70% of GD patients (2, 3). Patients with GO suffer from impaired visual function, facial disfigurement, and at worst, irreversible visual loss caused by corneal ulceration or dysthyroid optic neuropathy, which result in a poor quality of life and socio-economic status (4, 5). GO is a vexing autoimmune condition with both cellular and humoral immunities that form a sophisticated regulatory network, which leads to early orbital inflammation and late tissue remodeling (2, 4–6). Because of incomplete understanding of its precise pathogenesis, which partly results from the absence of suitable preclinical animal models, there is a lack of highly effective and well-tolerated therapies that target the most likely cause and glucocorticoids (GCs) are still the mainstay of treatment for active GO when inflammation is at peak (4, 5, 7, 8). Clinically, intravenous GC treatment has acceptable outcomes for most patients in the active phase. Nevertheless, a substantial number (20-30%) of active moderate-to-severe GO patients may not respond to GCs and adverse effects may occur after administration of high-dose or long-term GC use. Some patients may have disease progression despite GC treatment or relapse after steroid withdrawal (7, 8). Hence, a balance between benefits and risks of therapies for GO should be considered, which means developing more specific immunosuppressant strategies such as targeting T cells.

In the late 1980s, the role of T cell immunity was investigated in the orbital connective tissues of GO patients (9). Although thyroid-stimulating hormone receptor (TSHR) and its autoantibody play a major role in the pathological cascade of GO (2, 5), activation of humoral immunity, namely B cell immune responses, depends on defects in T cell immune modulation (10). The orbit is likely to have similar initial autoimmune reactions as those in the thyroid (5). It can be safely speculated that, among the various immune components that infiltrate the orbital connective tissues of GO patients, autoreactive T cells may act to establish and perpetuate the orbital inflammatory process. Recent studies have revealed that such disease-associated T cells include both T helper (Th) 1 (cytotoxic leaning) and Th2 (antibody leaning) subpopulations, and an emerging role of Th17 (fibrotic leaning) cells has also been implicated (6). The use of traditional non-specific immunosuppressants, such as cyclosporine that prevents interleukin (IL)-2 secretion by CD4^+^ T cells and mycophenolate that inhibits T cell proliferation by depleting guanosine-tri-phosphate, appear to be effective as a step-down from GCs to achieve stable efficacy in the long term (11). In view of the above-mentioned facts, phenotypic and functional analyses of orbit-infiltrating T cells may provide better insights into the pathogenesis of GO.

In this review, we provide a detailed overview of the dysregulated T cell immunity in GO pathology. We include the early data as well as the latest research to reflect the developing course of understanding GO orbital autoimmunity. A selected listing of recommended studies on T cell pathogenesis in GO is summarized in **Table 1**. We highlight the integral role of pathological T cells that have deleterious effects on fibrocytes and orbital fibroblasts (OFs), and describe the development of targeted therapies for GO in an effective and safe manner.

**Table 1:** Recommended Studies on T cell Pathogenesis in GO.

Reference	Study subjects		Main findings
**T cell immunity and TCR repertoires**			
Heufelder et al. (12)		Biopsies of thyroid glands, orbital connective tissues, pretibial skins, and PBMCs from two GD patients with both orbitopathy and dermopathy and two non-GO controls	Both orbital connective tissues and pretibial connective tissues were infiltrated by CD3^+^ T cells; Marked similarities of intrathyroidal, orbital, and pretibial TCR gene repertoires were found, which indicate apparent TCR restriction and T cell oligoclonality.
Pappa et al. (13)		Biopsies of EOMs from five early active GO patients, nine late stable GO patients, and 14 non-GO patients	CD4^+^ and CD8^+^ T cells and macrophages were significantly present in EOMs of active GO compared with both stable GO and controls; Increased HLA-DR expression on OFs, but not EOM fibres, was observed in both active and stable GO.
Rotondo Dottore et al. (14)		Biopsies of orbital connective tissues from 20 consecutive GO patients	A positive correlation was found between CD3^+^ T and CD20^+^ B cells infiltrating orbital connective tissues with GO clinical activity.
Wang et al. (15)		Biopsies of thyroid glands and PBMCs from six GD patients; PBMCs from 43 GO patients and 57 stable GD patients	A model for prediction of GO progression in GD cohort with high sensitivity and specificity.
Aniszewski et al. (16)		117 CD4^+^ T cell clones expanded from orbital connective tissues of 6 GO patients	Th1 immune response predominated in early active GO and Th2 immune response predominated in late stable GO.
**Effector T cell, OF, and fibrocyte interaction**			
Feldon et al. (17)		GO and control OFs; autologous T cells from PBMCs	Autologous T cells promoted the proliferation of GO OFs dependent on MHC class II and CD40-CD40L pathways.
Hwang et al. (18)		GO and control OFs	GO OFs expressed elevated levels of CD40 that could be further up-regulated by IFN-γ; CD40-CD40L combination led to IL-6, IL-8, and MCP-1 production in GO OFs; CD90^+^ GO OFs expressed more CD40 than CD90^-^ GO OFs.
van Steensel et al. (19)		Biopsies of orbital connective tissues from GO patients and controls; GO OFs	Mast cells, monocytes, and macrophages expressed increased levels of PDGF-A and PDGF-B in GO orbital connective tissues; PDGF-AB and PDGF-BB promoted proliferation and hyaluronan and IL-6 production by GO OFs.
Tsui et al. (20)		Biopsies of thyroid glands and orbital connective tissues; GO and control OFs; thyrocytes	TSHR levels were higher on thyrocytes than GO and control OFs; Differentiation of GO OFs, but not control OFs, into adipocytes led to increased TSHR expression; IGF-1R levels were higher on GO OFs than control OFs; TSHR and IGF-1R colocalized to the perinuclear and cytoplasmic areas of both GO OFs and thyrocytes.
Cao et al. (21)		GO and control OFs	CD40-CD40L combination led to the synthesis of hyaluronan and PGE_2_ in GO OFs; PGE_2_ production in GO OFs was caused by increased expression of PGSH-2 at both transcriptional and translational levels regulated by IL-1α expression
Koumas et al. (22)		GO OFs; myometrial fibroblasts	CD90^+^ myometrial fibroblasts and GO OFs were capable of myofibroblast differentiation by TGF-β or platelet concentrate supernatant treatment; CD90^-^ myometrial fibroblasts and GO OFs were capable of lipofibroblast differentiation by 15-deoxy-Δ^12,14^-PGJ_2_ or ciglitazone treatment.
Antonelli et al. (23)		Sera from consecutive subjects including 60 GD patients, 60 GO patients, and 60 controls; GO thyrocytes, OFs, and induced preadipocytes; Control fibroblasts and induced preadipocytes from dermal tissues of the same patients	CXCL10 was higher in GD and GO patients than controls; CXCL10 was significantly higher in active GO patients than inactive GO patients; IFN-γ and TNF-α synergistically induced CXCL10 production in GO thyrocytes, OFs, and preadipocytes, which was suppressed by PPAR-γ agonist.
Antonelli et al. (24)		GO thyrocytes, OFs, and induced preadipocytes; Control fibroblasts and induced preadipocytes from dermal tissues of the same patients	IFN-γ and TNF-α synergistically induced CXCL9 and CXCL11 production in GO thyrocytes, OFs, and preadipocytes, which was suppressed by PPAR-γ agonist.
Han et al. (25)		GO and control OFs	IFN-γ and IL-4 attenuated IL-1β-provoked PGE_2_ production by suppressing PGHS-2 gene promoter activity but enhanced IL-1β-initiated hyaluronan production by up-regulating hyaluronan synthase-2 gene expression in GO OFs.
Han et al. (26)		GO and control OFs	IFN-γ and IL-4 attenuated IL-1β-induced TIMP-1 production by suppressing TIMP-1 gene promoter activity in GO OFs.
Huber et al. (27)		Whole blood from 216 GD patients and 368 healthy controls	rs2201841 was strongly associated with GO development, especially AA and CC genotypes of *Il23r*.
Douglas et al. (28)		Biopsies of orbital connective tissues; PBMCs from 70 GD patients (including 51 GO patients) and 25 healthy controls; GO and control OFs; thyrocytes; fibrocytes	CD34^+^CXCR4^+^Collagen I^+^TSHR^+^ fibrocytes were increased in PBMCs of GD patients; TSH induced fibrocytes to produce IL-6 and TNF-α; Increased fibrocytes were found in orbital connective tissues of GO patients.
Gillespie et al. (29)		PBMCs from 31 GO patients and 19 healthy controls; GO OFs; GO and control fibrocytes	Fibrocytes expressed higher levels of TSHR than GO OFs; GO fibrocytes expressed higher levels of TSHR than control fibrocytes; TSH or M22 greatly stimulated the production of various cytokines and chemokines such as IL-8, RANTES, and MCP-1 in both GO and control fibrocytes.
Fang et al. (30)		Biopsies of orbital connective tissues; PBMCs from 34 GO patients and 36 healthy controls; GO and control OFs; *in vitro*-differentiated Th17 cells	GO peripheral Th17 cells produced IFN-γ and IL-22 and were related to clinical activity score; IL-17A enhanced TGF-β–induced fibrosis in CD90^+^ OFs but inhibited 15-deoxy-Δ^12,14^-PGJ_2_–induced adipogenesis in CD90^-^ OFs; Th17 cells stimulated proinflammatory cytokine expression of GO OFs and GO OFs promoted Th17 cell differentiation by PGE_2_ production.
Fang et al. (31)		21 GO orbital connective tissues and 38 control orbital connective tissues; CD34^+^ GO OFs; *in vitro*-differentiated Th17 cells	GO orbital microenvironment was composed of T cells, B cells, natural killer cells, dendritic cells, macrophages, plasma cells, and CD34^+^ OFs; Orbit-infiltrating Th17 cells displayed a Th1-like phenotype and expressed high levels of IL-1R and IL-23R; CD34^+^ OFs enhanced IL-1R and IL-23R expression on Th17 cells by PGE_2_-EP2/EP4-cAMP signaling.
Fang et al. (32)		PBMCs from 16 active and 14 stable GO patients and 20 healthy controls; GO and control fibrocytes; *in vitro*-differentiated Th17 cells	IL-17A stimulated cytokine production in both GO and control fibrocytes; Autologous Th17 cells promoted inflammatory and antigen-presenting functions of GO fibrocytes; GO fibrocytes enhanced Th17 cell phenotype and recruited Th17 cells by MIP-3 and CCR6 combination.
Fang et al. (33)		Biopsies of orbital connective tissues; Sera and PBMCs from consecutive subjects including 37 GO patients, 38 GD patients, and 32 healthy controls	Increased CXCR3^+^ IFN-γ–producing Th17.1 cells were positively correlated with GO activity and associated with the development of very severe GO; In GC-resistant, very severe GO patients, CXCR3^+^ IFN-γ–producing Th17.1 cells remained at high levels in blood and orbital connective tissues, which were positively correlated with elevated triglycerides.
Fernando et al. (34)		GO OFs; GO and control fibrocytes	TSH and M22 induced IL-23, but not IL-12, expression in fibrocytes, while they induced IL-12 production in GO OFs; The shift from IL-23 expression in fibrocytes to that of IL-12 in CD34^+^ GO OFs was regulated by Slit2.
**GO animal model**			
Moshkelgosha et al. (35)		hTSHR-A subunit plasmid-immunized BALB/c mice	TSHR was the pathogenic antigen in GO; Interstitial inflammation of extraocular muscles with CD3^+^ T cells, F4/80^+^ macrophages, and mast cells, accompanied by glycosaminoglycan deposition was observed in murine orbits.
Zhang et al. (36)		hTSHR-A subunit-expressing adenovirus-immunized BALB/c mice	Fibrosis and adipogenesis accompanied by CD4^+^ T cell infiltration were seen in murine periorbital fat tissues; Increased frequencies of Th1 cells and decreased frequencies of Th2 cells and regulatory T cells were shown in the splenocytes of GO mice.
Masetti et al. (37)		hTSHR-A subunit plasmid-immunized BALB/c mice	*Bacteroides* and *Bifidobacterium* counts were more abundant in mice in Center 1, while *Lactobacillus* counts were more abundant in mice in Center 2; Significantly higher yeast counts were found in Center 1 TSHR-immunized mice; A significant positive correlation was found between the presence of *Firmicutes* and orbital adipogenesis in Center 2 TSHR-immunized mice.

## CD4^+^ and CD8^+^ T Cell Immunities in GO

The first issue is whether cellular immunity is involved in GO inflammation. In an early study, Heufelder et al. reported the presence of CD3^+^ cells that represent total T cells in orbital and pretibial connective tissues from two GD patients with both orbitopathy and dermopathy (12). The results provide evidence of T cells infiltrating the inflamed orbit. Phenotypic analysis of four peripheral blood mononuclear cell (PBMC) samples from four severe GO patients revealed the main subtype as CD4^+^ T cells (CD4/CD8 ratios 1.9-2.5), which was similar to the phenotypes of four control PBMC samples, whereas their corresponding orbital connective tissue-derived T cell lines had equal amounts of CD4^+^ and CD8^+^ T cells (CD4/CD8 ratios 0.9-1.2) (38). The ratios of CD4/CD8 were unchanged in 153 GO T cell clones cultivated from the four orbital T cell lines and 166 and 236 T cell clones cultivated from the four PBMC samples of GO patients and control subjects, respectively (38). The relatively low ratios of CD4/CD8 in orbital connective tissue-derived T cell lines and clones indicate that there is a disorder of cellular immune function in GO orbits. Grubeck-Loebenstein et al. established and characterized six T cell lines from orbital connective tissues of two severe GO patients and found they were predominantly CD8^+^CD45RO^+^ T cells (77%-96%) (39). The above two studies imply that a cytolytic T cell immunity triggered by CD8^+^ T cells may contribute to orbital inflammation in GO in a major histocompatibility complex (MHC) class I dependent manner. But the results cannot tell whether there exists a more efficient and unique antigen-presenting process to activate orbit-specific T cells. Stover et al. screened 64 orbital connective tissue-derived T cell clones expanded from two GO patients and reported an obvious predominance of the CD4^+^ T cell population (CD4/CD8 ratio 8.2) that contrasted with six PBMC samples (CD4/CD8 ratio 2.1) (39). In another study, the same research group analyzed 10 of 17 T cell lines derived from orbital connective tissues of six severe GO patients and found mainly CD4^+^ T cells (six of 10 strains) with a similar CD4^+^/CD8^+^ T cell distribution (40). The studies supporting the role of CD4^+^ T cells suggest an MHC class II pathway primed by a specialized antigenic determinant within the thyroid and at the involved orbital connective tissues. Pappa et al. investigated the extraocular muscles (EOMs) of 10 GO patients who underwent corrective strabismus surgery and examined six EOM-derived T cell lines from four patients. Five were CD4^+^CD45RO^+^ T cells (85%-97%) and CD8^+^ T cells (68%) were dominant in only one strain. The same status was found in the four corresponding PBMC samples (three were mostly CD4^+^ cells (89%-98%)) of each patient. They further reported detectable T cell receptor (TCR) gene expression in 10 out of 12 EOMs collected from the other five patients and in all five EOMs collected from three control subjects (41). The discrepancy of CD4^+^ and CD8^+^ T cell subsets in the above findings may lie in the small number of patients, the heterogeneity of patients involved in the different studies, and the different research methods. Notably, the T cell lines or clones in the above studies were cultured tissue- or peripheral blood-derived T cells expanded for several days to weeks, which may affect the initial status of these T cells to a certain extend. For example, CD8^+^ T cells may have more rapid expansion and CD4^+^ T cells gradually die during culture. Förster et al. established 18 T cell lines from orbital connective tissues of six severe GO patients and reported that 10 were predominantly the CD4^+^ phenotype, whereas three were mostly CD8^+^ cells (42). Intriguingly, in their study, even two independent T cell lines derived from the same patient had distinct T cell phenotypes (CD4 or CD8). This indicates that both CD4^+^ and CD8^+^ T cells are involved in GO pathogenesis. However, the phenotypic analysis was also based on T cell lines cultured *in vitro*. Therefore, direct *in vivo* T cell examination is needed to avoid biases and better reflect the real orbital immunity in GO inflammation.

Subsequently, an *in situ* study by immunohistochemistry demonstrated that both CD4^+^ and CD8^+^ T cells had infiltrated the EOMs in early active GO, which were much less evident in late inactive GO and control subjects (13). A recent study examined 26 GO patients and seven control subjects by immunohistochemistry, which showed that TCR expression was strong and diffuse in severe patients, although the orbital TCR detectable rate was similar in both active severe and inactive mild GO. Active severe GO patients had a higher CD3 detectable rate compared with inactive mild GO patients. Additionally, no expression of TCR or CD3 was found in control orbits (43). These data support the idea that GO orbital connective tissues are variably infiltrated by lymphocytes during active disease when medications are more effective than in the inactive disease.

We used flow cytometric analysis and found no differences in the frequency of circulating CD4^+^ and CD8^+^ T cells or the ratios of CD4/CD8 between GO patients and control subjects (44). In agreement with the above immunohistochemistry studies, infiltrated CD4^+^ and CD8^+^ T cells extended throughout the orbital connective tissues of GO patients, especially in the active phase, compared with control subjects (44, 45). Rotondo Dottore et al. confirmed that the total number of orbit-infiltrating T cells was correlated positively with the GO clinical activity score in simple and multiple linear regression models (14). Studies in GO murine models also supported T cell-mediated inflammation in the orbit *in vivo*. CD3^+^ total T cells were found to infiltrate into the orbital muscles and periorbital tissues of human (h) TSHR-A subunit plasmid-immunized BALB/c mice (35, 46). The same phenomenon was found in mouse TSHR-A subunit plasmid-immunized BALB/c mice (47). Intriguingly, increased CD4^+^ T cell subsets were reported in periorbital fat of SKG mice after intraperitoneal administration of zymosan A compared with wild type mice (48). A recent study used an adenovirus that expressed the hTSHR-A subunit to induce GO in BALB/c mice and also observed CD4^+^ T cell infiltration in periorbital fat tissues (36). Collectively, these data shed light on the presence and type of T cells in GO, which suggest a complex inflammatory microenvironment in the orbit.

## Self-Reactive T Cells Directed Against OFs

The second issue is whether T cells in GO recognize autoantigens, i.e., a primary GO immune response leads to the development of antigen-specific T cell responsiveness and clonal proliferation in the orbit. This will determine whether T cell immunity is specifically directed against orbital antigens. Heufelder et al. reported that in the two GD patients with both orbitopathy and dermopathy the vast majority of TCRs in the orbital and pretibial connective tissues were αβ chains and not γδ chians (12). Although expression of a broad spectrum of both TCR Vα and Vβ genes was observed in the PBMCs of patients, marked restriction of TCR Vα and Vβ gene expression was found in thyroid glands and orbital and pretibial connective tissues compared with PBMCs. Furthermore, thyroid, orbital, and pretibial tissues from two control subjects did not express restricted TCR transcripts (12). These data imply the potential GO-specific oligoclonal expression of the TCR gene repertoire. To further characterize the limited variability of antigen receptors on extrathyroidal T cells in GO, Heufelder et al. examined the TCR V gene repertoire *in situ* in orbital connective tissues and EOMs from eight early severe GO patients and observed apparent TCR Vα and Vβ gene restriction compared with matched PBMCs. Loss of TCR gene restriction was observed in four late GO patients and no TCR gene restriction was found in samples from three non-GO control subjects (49, 50). These findings suggest that oligoclonality of T cell immunity may be lost during GO, which indicates that antigen specificity of orbit-infiltrating T cells occurs in the early active phase of GO. This is important because an early adaptive immune response implies organ-specific autoimmunity in orbital connective tissues independent of the thyroid. Development of diversity or polyclonality of the TCR gene repertoire indicates that orbital inflammation is at the burnout stage. Heufelder summarized data from three severe active GO patients with GD and dermopathy and reported not only marked TCR restriction, but also several conserved junctional motifs shared by T cells in the orbit, thyroid, and pretibial tissue despite obvious heterogeneity of the TCR genes in each patient (12, 51). This highlights the presence of certain oligoclonal T cell populations stimulated by the analogous antigenic determinants shared by the thyroid and the involved extrathyroidal compartments. A recent interesting study proposed a novel TCR clonal expansion and chaos score to predict GO development in GD by characterizing complementarity determining region 3 of the TCR Vβ gene repertoire in PBMCs, which indicates specific GO TCR signatures distinctive from GD (15). These selected TCR-bearing T cells are self-reactive and recruited to the orbit at GO attack, which lead to orbital inflammation.

The next issue is unraveling the specific cell type or protein that triggers GO self-reactive T cell expansion. Genetic immunization with mouse TSHR-A subunit breaks self-tolerance and induces GO-like pathology in BALB/c mice (47). Splenic T cells from BALB/c mice that have received hTSHR-A subunit prepared as a maltose-binding protein fusion induce orbital pathology in naïve recipient BALB/c mice marked by the presence of CD3^+^ total T cells (52). Furthermore, splenic T cells from hTSHR-A subunit plasmid-primed GO BALB/c mice show proliferative responses to purified TSHR antigen (53). These data from animal models provide a clue to potential TSHR-specific T cell responses that may also occur in the GO patient orbit. Arnold et al. reported occasional proliferation responses to EOM antigens in 10 circulating T cell lines from 10 severe GO patients. Additionally, these T cells hardly produced interferon (IFN)-γ under EOM antigen stimulation (54). Similarly, in the *in vitro* model presented by Grubeck-Loebenstein et al., six T cell lines from orbital connective tissues did not proliferate in response to EOM antigen stimulation, but all had apparent proliferation after autologous OF treatment (39). In the *in vitro* model of Otto et al., the established 17 orbital T cell lines responded significantly to autologous orbital connective tissue proteins (6-10 and 19-26 kDa). A similar phenomenon was seen in most GO PBMCs that were more sensitive to autologous proteins from OFs than myoblasts. Moreover, orbital T cell lines hardly responded to allogeneic orbital proteins (40). Conversely, the authors demonstrated that 18 established T cell lines were barely able to respond to TSHR (2/18), thyroidal peroxidase (2/18) or thyroglobulin (none) (42). The results suggest the primary antigen role of TSHR and antigen-specific T cell clones in GO patients. However, the relatively low proliferation rate is confusing. It is important to note that although irradiated autologous PBMCs were added as feeders to help T cell to clone in these two studies, the antigen-induced T cell-specific proliferative response is acted in an antigen-presenting cell (APC)-dependent manner. The same research group used PBMCs from 16 GO patients and 12 controls and confirmed that incubation of GO PBMCs with OFs from the same patients led to marked T cell proliferation compared with control OFs. Similarly, compared with control OFs, GO OFs also had increased proliferation responses to stimulation by autologous PBMCs (55). This implies that OFs express GO autoantigens, and we hypothesize that GO OFs may function as facultative APCs to stimulate the proliferation of antigen-specific T cells, which has been confirmed by the fact that autologous T cells also stimulate the proliferation of GO OFs, but not eyelid-derived fibroblasts, *via* MHC class II and CD40-CD40 ligand (CD40L) signaling (17). We and other groups have shown that GO orbital connective tissues express higher gene and protein levels of MHC II and CD40 than control subjects (18, 30, 43, 56). Moreover, MHC II^+^ cells and CD40^+^ cells are local fibroblast-shaped cells and invading mononuclear cells such as macrophages in orbital connective tissues (18, 56). Even in stable GO, orbital connective tissues are activated to persistently express MHC II (56). Similarly, murine OFs derived from hTSHR-A subunit plasmid-primed BALB/c mice showed strong expression of CD40, TSHR, and insulin-like grow factor 1 receptor (IGF-1R) (57). Taken together, these findings have revealed sensitized and orbital connective tissue-specific T cells in circulation and the orbit of GO patients and provided evidence for self-reactive T cell populations directed against OFs.

## Primary Immune Reaction in the Orbit

**Figure 1:**
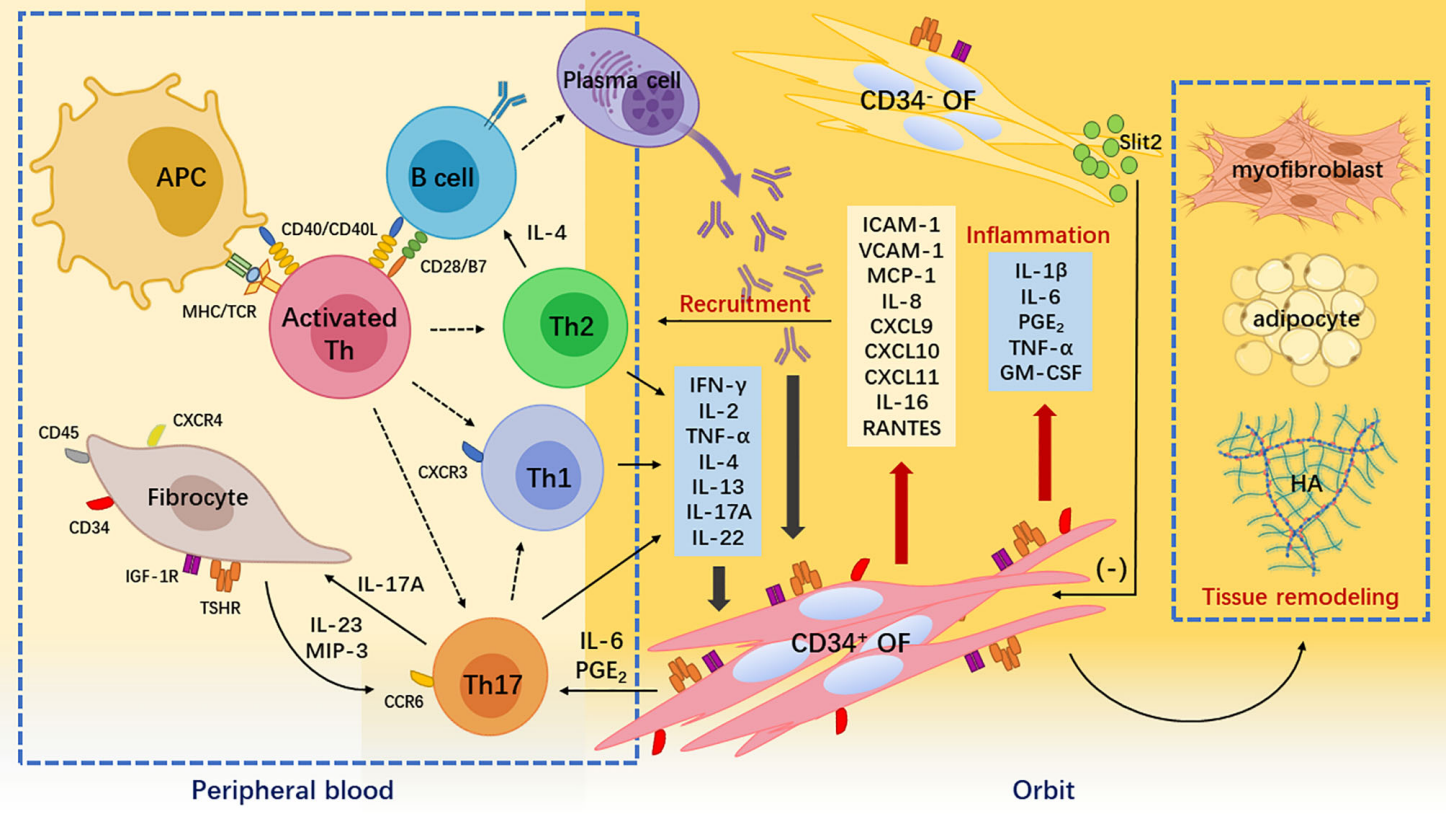
Pathogenesis of Graves’ orbitopathy. Breakdown of self-tolerance to thyroid-stimulating hormone receptor (TSHR) leads to the recognition of TSHR epitopes by antigen-presenting cells and B cells. They process the TSHR peptides to activate naïve T helper (Th) cells. Activated and expanded naïve Th cells differentiate into different subsets including interferon (IFN)-γ-producing Th1 cell, interleukin (IL)-4-producing Th2 cells, and IL-17A-producing Th17 cells. These cytokines together with autoantibodies produced by plasms cells derived from self-reactive B cells stimulate orbital fibroblasts (OFs), thereby initiating inflammatory responses in the orbit. IFN-γ is cytotoxic, IL-4 helps B cell expansion and autoantibody class switching, and IL-17A is proinflammatory and profibrotic. Meanwhile, peripheral CD34^+^ fibrocytes infiltrate orbital connective tissues and transition into CD34^+^ OFs. Upon IFN-γ and IL-17A stimulation, these CD34^+^ cells not only robustly produce chemokines such as C-X-C motif ligand 9/10/11 attracting C-X-C chemokine receptor 3^+^ Th1 cell and macrophage inflammatory protein-3 attracting C-C chemokine receptor 6^+^ Th17 cells but also secrete a large amount of cytokines such as IL-1β and prostaglandin E_2_ that exacerbate orbital inflammation. CD34^+^ OFs ultimately synthesize hyaluronic acid and differentiate into adipocytes or myofibroblasts that cause orbital tissue remodeling. The orbital native residential CD34^-^ OFs express immunomodulatory molecules such as Slit2 to restrain the activities of the infiltrating CD34^+^ OFs.

The third issue is how T cells and OFs communicate with each other, which causes a series of pathophysiological changes in the GO process. Evidence for both T and B cells infiltrating GO orbital connective tissues was shown as early as 2012 (58). Many other *in situ* immunohistochemistry studies have demonstrated the presence of CD4^+^ T cells (44, 45, 56, 59), CD20^+^ B cells (14, 60, 61), CD14^+^ monocytes (19, 56), CD68^+^ macrophages (19, 59, 62), and CD117^+^ mast cells (19, 63) as the main invading immune components in the GO orbit. Using single cell sequencing analysis, we showed that various genes are expressed in GO orbital connective tissues, which can be classified into six independent cell types: APCs, lymphocytes including T and B cells, OFs, adipocytes, endothelial cells, and myocytes (31). This indicates an extremely complicated local orbital microenvironment, in which infiltrating immune cells and non-immune stromal cells interact with each other. The presence of APCs in the orbit further supports the idea that a primary GO autoimmune reaction occurs within the extrathyroidal compartment, although it resembles the process in the thyroid. OFs that express TSHR and IGF-1R (20, 31) detect danger signals to guide the property and intensity of the GO-adaptive immune response (64, 65). TSHR has been recognized as an autoantigen, but might not be the only one that activates GO self-reactive T cells. More work is needed to explain why GO occurs in patients with Hashimoto’s thyroiditis with no evident TSHR autoimmune reactivity (2, 8). Although IGF-1R might be another autoantigen, GO pathology does not involve directly stimulating IGF-1R antibodies (5, 8). Serum antibodies against the IGF-1R are greater in GD patients, regardless of the presence of GO, suggesting a less pathogenic role of IGF-1R antibodies in GO (66, 67). In fact, a functional and physical crosstalk between TSHR and IGF-1R seems to be more important (5, 8). Defects in immune modulation lead to the breakdown of self-tolerance to thyroid or orbital connective tissues. Then, antigen-presenting cells (OFs themselves, fibrocytes, B cells, macrophages, or dendritic cells in the vicinity) recognize the exposed TSHR epitopes on the OF surface and then process and present the TSHR peptides to T cells, which leads to T cell clonal expansion and migration into orbital connective tissues (5, 6, 64). It should be noted that self-reactive B cells are likely to participate in GO antigen-presenting process. In recent onset GD patients, autoreactive B cells in PBMCs expressed CD86 and no longer appeared anergic, which indicates the activation and differentiation of B cells into plasma cells, leading to autoantibody production (68). Hence, it is reasonable to postulate that the same immune response occurs in orbital connective tissues in GO, where high levels of TSHR autoantibodies are detected in the sera of patients and may precede the onset of eye disease. A vital role of rituximab in the treatment of GO may lie in the blockade of antigen-presenting function of these activated self-reactive B cells (69). In addition, the over-reactive immune process has many other complicated mechanisms including thymic and peripheral T cell deletions and T cell anergy (5, 6, 70). Activated T cells provide the second signal for self-reactive B cell activation *via* the interaction of CD40L on the T cell surface with CD40 on the B cell surface. Moreover, the combination of B7 on the B cell surface and CD28 on the T cell surface provides the second signal for further activation of self-reactive T cells (5, 64, 71). Autoantibodies against TSHR are produced by plasma cells differentiated from activated B cells and autoantibody class switching (IgM to IgG and IgE) is aided by IL-4 secreted by activated T cells (mainly Th2 cells) (5, 64, 71). Autoantibodies, including stimulating, neutralizing, and blocking IgG (72), target the TSHR on OFs, which may promote cytokine and chemoattractant production, deposition of extracellular matrix (ECM) such as hyaluronan, and pathological OF differentiation into adipocytes and myofibroblasts (73). Potential cross-talk of TSHR with IGF-1R on OFs helps to augment the expression of inflammatory molecules and hyaluronan synthesis (74, 75). The above pathological processes are largely due to the cell contact between OFs and T cells and cytokines produced by various T cell types (**Figure 1**).

**Figure 2:**
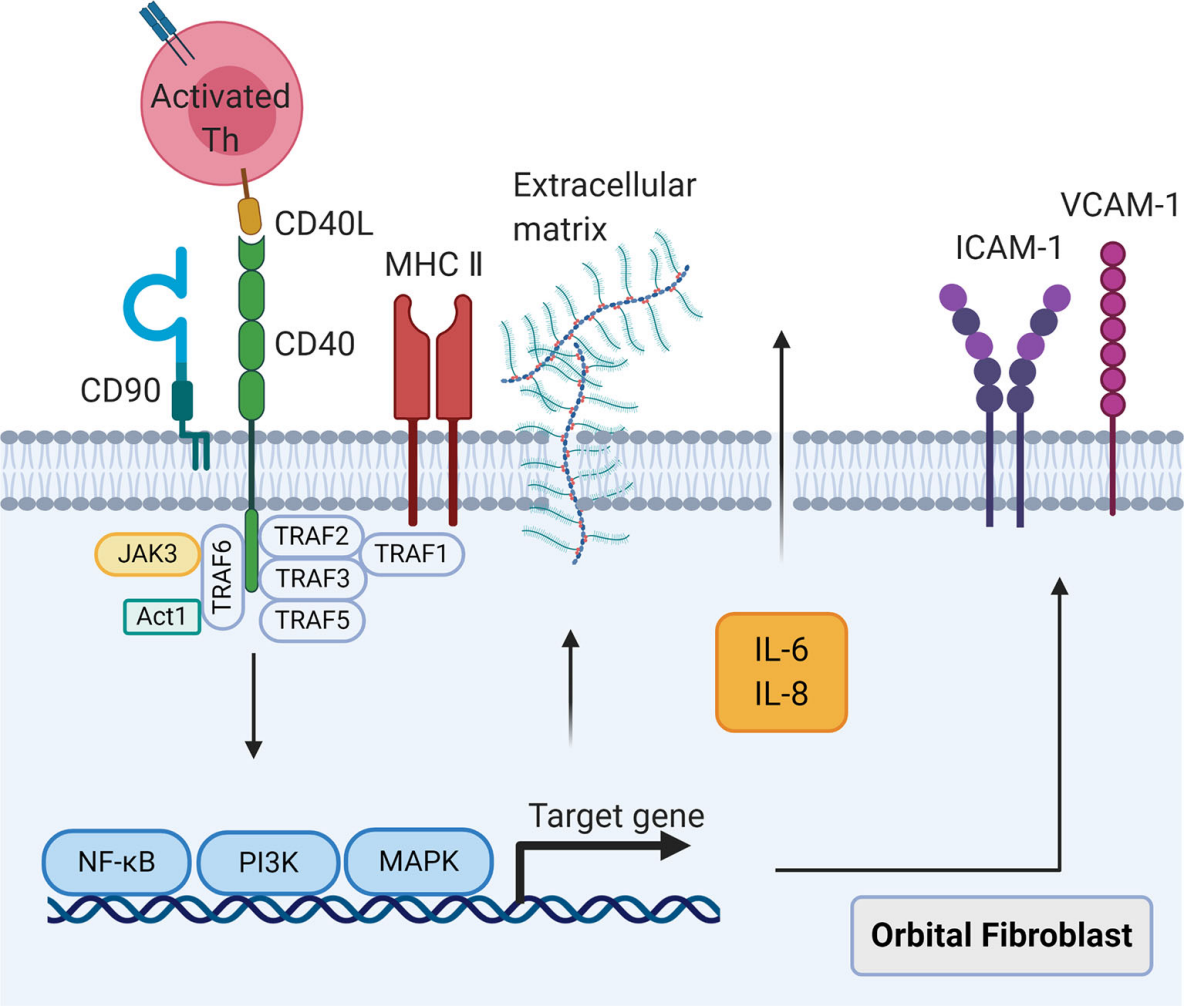
The CD40-CD40 ligand signaling in orbital fibroblasts. When CD40 ligand on self-reactive T cells combines with CD40 on orbital fibroblasts, the nuclear translocation of nuclear factor-κB, mitogen activated protein kinases, and phosphatidylinositol-3-kinase signaling pathways will be activated, leading to the synthesis of cytokines interleukin-6 and interleukin-8, costimulatory molecules intercellular adhesion molecule-1 and vascular cell adhesion molecule-1, and extracellular matrix.

An important intercellular communication in GO is CD40-CD40L signaling (**Figure 2**). CD40 is a mitogenic receptor that belongs to the tumor necrosis factor (TNF)-α receptor superfamily (76). CD40 is constitutively expressed by human fibroblasts derived from different tissue sources including OFs (18, 76), which facilitates fibroblast proliferation (76). GO OFs express elevated CD40 at gene and protein levels compared with control OFs (18, 77). When delineated by the cell surface marker CD90, CD90^+^ GO OFs had considerably greater CD40 expression than that on CD90^-^ subsets as well as both control OF subsets (18). The combination of CD40 on OFs with CD40L on T cells leads to the three following pathological effects: (1) The release of inflammatory cytokines that induce acute and chronic orbital inflammation. Activation of GO OFs by CD40 engagement elevates IL-6 and IL-8 protein levels comparable with those produced by CD40-activated control OFs (77). Additionally, GO OFs primed with IFN-γ appear to be more responsive to CD40 activation than control OFs with regard to macrophage chemoattractant protein-1 (MCP-1) expression (18). Intriguingly, overproduction of IL-6 and IL-8 has been observed in CD90^+^ GO OFs compared with CD90^-^ GO OFs after priming with IFN-γ (18). Conversely, CD40-CD40L signaling stimulates relatively low IL-6 and IL-8 production in both control OF subsets even when pre-incubated with IFN-γ (18). Hence, the higher expression of CD40 on CD90^+^ GO OFs may be critical to generate IL-6 and IL-8 in response to CD40L. Moreover, time-dependent secretion of prostaglandin (PG) E_2_ from GO OFs induced by CD40 engagement has been attributed to the up-regulation of IL-1α production, which enhances the expression of prostaglandin endoperoxide H synthase-2 (PGSH-2 or COX-2) at both transcriptional and translational levels (21). (2) Up-regulation of adhesion molecules promotes immune cell recruitment to orbital connective tissues. GO orbital connective tissues expressed higher levels of intercellular adhesion molecule-1 (ICAM-1) and vascular cell adhesion molecule-1 (VCAM-1) compared with control subjects (30). The gene and protein expressions of ICAM-1 (78), VCAM-1, and E-selectin (79) were increased by CD40 activation in both GO and control OFs in dose- and time-dependent manners, with a more obvious effect on the former. Moreover, sphingolipids were induced in GO OFs, but not control OFs, which attracted T cells to migrate (80). (3) Synthesis of hyaluronan leads to the edema of orbital connective tissues and late-stage tissue remodeling. These phenomena are independent of PGE_2_ synthesis in GO OFs (21). Signal transductions for the pathways involved in the CD40-CD40L combination mentioned above mainly include nuclear translocation of nuclear factor-κB, mitogen-activated protein kinases, and phosphatidylinositol-3-kinase. Further studies are needed to examine in more depth the cognate interactions between GO OFs and infiltrating T cells *via* CD40-CD40L communication.

Furthermore, T cell-OF interaction triggers the differentiation of OFs into adipocytes (8). Feldon et al. reported that GO orbital connective tissues and GO OFs highly expressed peroxisome proliferator-activated receptor (PPAR)-γ mRNA and proteins (81). In an autologous periphery T cell-OF coculture system, activated T cells drove the differentiation of GO OFs into orbital adipocytes. These activated T cells not only expressed up-regulated levels of PGSH-2 but also synthesized PGD_2_ and related PGJ_2_ that are PPAR-γ ligands (81). This PPAR-γ-dependent adipogenic process of GO OFs provides evidence for how inflammation-provoking T cells regulate adipogenesis of orbital connective tissues and an interesting clue to the many faces of T cell immunity in GO.

## Paradigm of Th1/Th2 Immune Responses

Previous studies have shown that both Th1 and Th2 cell subsets are involved in GO autoimmunity. In a study by De carli et al., 153 orbital T cell clones expanded from four severe GO patients exhibited remarkably high proportions of both CD4^+^ and CD8^+^ T cell phenotypes with a Th1-like cytokine profile including IFN-γ (82% in CD4; 88% in CD8), IL-2 (79% in CD4; 81% in CD8), and TNF-α (90% in CD4; 88% in CD8), but not IL-4 (4% in CD4; 0% in CD8) or IL-5 (1% in CD4; 0% in CD8), compared with T cell clones expanded from PBMCs of both GO patients and control subjects (38). Förster et al. examined cytokine gene expression in 18 orbital T cell lines from six severe GO patients and detected expression of Th1 cytokine genes *Ifng* (10/18), *Tnfa* (12/18), and *Il2* (17/18) as well as Th2 cytokine genes *Il4* (12/18) and *Il5* (17/18). Other expressed cytokine genes were *Il6* (13/18), *Il10* (4/18), and *Tnfb* (15/18) (42). Using orbital T cell clones expanded from three severe GO patients, Yang et al. observed expression of *Ifng* and *Il2* in eight out of 14 CD4^+^ T cell clones and four out of six CD8^+^ T cell clones. The authors also assessed cytokine secretion in 38 CD4^+^ and 10 CD8^+^ strains, including the T cell clones for gene expression examination, and reported detectable levels of IFN-γ in most T cell clones (36/38 in CD4; 9/10 in CD8), of which some secreted IL-2 (8/36 in CD4; 5/10 in CD8). No Th2 cytokine gene profile and only three IL-4-secreting and five IL-10-secreting T cell clones were found (82). These results indicate that the great majority of orbit-infiltrating T cells express a Th1-like cytokine profile at both transcriptional and translational levels. In a study by Pappa et al., nine EOM-derived T cell lines from four GO patients were all positive for Th1 cytokine IFN-γ and IL-2. Other tested cytokines included TNF-β (5/9) and transforming growth factor (TGF)-β (9/9). They also found that Th2 cytokine IL-4 was positive in three out of five examined T cell lines (among the nine T cell lines) and IL-10 was positive in four out of five. However, the detectable rates of cytokines genes *Il1a*, *Il2*, *Il4*, *Il6*, *Il8*, *Il10*, *Il5*, and *Tnfa* varied among another 12 different EOMs of a further five patients. Expression of *Ifng*, *Il13*, *Il1b*, and *Il12p40* was not detected in these EOMs. *Il6* and *Il8* were the only cytokine genes expressed in two out of five EOMs from three control subjects (41). It should be considered that gene and protein expressions are not complete coincident. Furthermore, apart from the technical problems related to the lymphocyte number and sample size, the various pre-surgery treatments that each patient had received and whether T cell clones were consecutively included or selected from independent patient cohorts will introduce biases and affect the results.

An important study by Aniszewski et al. examined cytokine production of 57 CD4^+^ T cell clones expanded from six GO patients and explicitly showed that T cell clones from recent onset GO (less than 2 years) mainly produced IFN-γ (47%) compared with IL-4 (23%), whereas those from remote onset GO (more than 2 years) dominantly produced IL-4 (75%) compared with IFN-γ (0%) (16). These findings suggest that the Th1 immune response may predominate in early active GO and the Th2 immune response is likely to play a role in late inactive GO. Unfortunately, all T cell strains examined were cultured and expanded *in vitro* for many days and only four T cell clones were matched for late GO, which drew criticism of the study design in which conclusions were made on the basis of data that may result in inaccurate estimates of T cell populations that invaded *in situ* within the orbit. Additionally, short duration GO is not exactly the same as active GO and longer duration GO does not mean that orbital inflammation is apparently absorbed.

By directly investigating the cytokine gene expression of EOMs *in vivo* from 14 GO patients and orbital connective tissues from 29 GO patients, expression of *Ifng* (13/14), *Tnfα* (5/14), *Il1b* (8/14), and *Il6* (9/14) was mainly detected in EOMs, while they were less often expressed in orbital connective tissues in which *Il4* (7/29) and *Il0* (11/29) were more frequently expressed (83). Notably, the mean GO duration of the patients involved in the study was 2 years, which may account for the lower expression of *Ifng* and relatively higher expression of *Il4* in orbital connective tissues. Additionally, we cannot neglect the fact that all patients had undergone orbital irradiation before tissues were obtained and half had received high-dose GCs. Wakelkamp et al. investigated cytokine gene expression in orbital connective tissues from 17 GO patients, of whom six had untreated active disease and underwent emergency decompression surgeries. The other 11 patients were in the inactive phase and underwent rehabilitative surgeries. Compared with inactive GO patients, active GO patients had increased expression levels of *Il1b*, *Il6*, *Il8*, and *Il10*. Expression of Th1 cytokine genes *Ifng*, *Il2*, and *Il12p40* was also higher in active orbital connective tissues. However, expression of Th2 cytokine genes *Il5* and *Il13* was comparable in both patient groups and *Il4* expression was not found in the study (84). These data imply the importance of the Th1, but not Th2, immune response and many other proinflammatory cytokines in the autoimmune environment in the active GO orbit, although the gene expression results need to be supported at the protein level. Furthermore, the source of the examined cytokines cannot be identified in the bulk sequencing data.

Many recent studies have confirmed that orbital connective tissues from GO exhibit strong immunostaining for IFN-γ in the infiltrating cells, especially in the active phase (33, 45, 61). Using *in vivo* flow cytometric analysis, we demonstrated an increased frequency of CD3^+^CD8^-^ IFN-γ-producing T cells in both GO peripheral blood and orbital connective tissues compared with control subjects (31, 45). In hTSHR-A subunit plasmid-primed GO BALB/c mice, splenic T cells secreted IFN-γ (53). An increased frequency of CD4^+^ IFN-γ-producing T cells derived from splenocytes has also been observed in hTSHR-A subunit-expressing adenovirus-immunized GO BALB/c mice (36). In an SKG murine model of GO, expression of *Ifng*, *Tnfa*, and *Il2* was augmented in periorbital tissues and their protein levels were elevated in sera compared with wild type mice (48).

**Figure 3:**
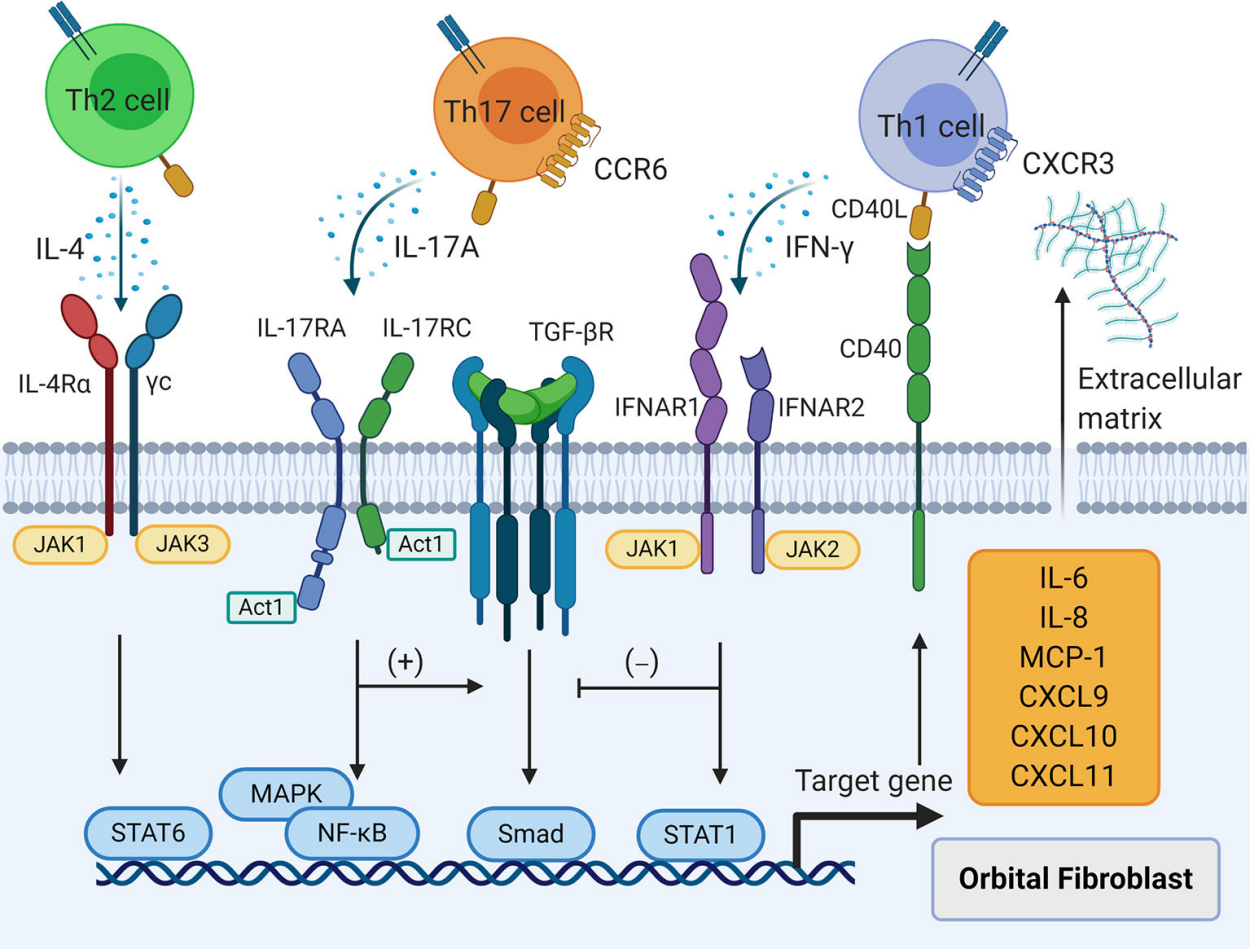
The effects of T cell cytokines on orbital fibroblasts (OFs). T helper (Th) 1 cytokine interferon-γ, Th2 cytokine interleukin (IL)-4, and Th17 cytokine IL-17A result in the production of IL-6, IL-8, macrophage chemoattractant protein-1, C-X-C motif ligand 9/10/11, and extracellular matrix in OFs. Interferon--γ interferes with transforming growth factor-β induced fibrosis in OFs while IL-17A strengthens this process.

However, the pathological effects of IFN-γ on OFs are not fully understood (**Figure 3**). An essential function of IFN-γ in GO is acting as the “sinister partner” of CD40-CD40L signaling to exacerbate orbital inflammation. IFN-γ not only up-regulates CD40 on human fibroblasts derived from lung, skin, and gingiva, but also shifts fibroblasts to the G0/G1 phase of the cell cycle (76). Expression of CD40 was further augmented by IFN-γ in both GO and control OFs regardless of CD90 expression (18, 77, 85). Additionally, MHC II expression was increased in both GO and control OFs after IFN-γ stimulation (17, 77, 85) with a more potent effect on CD90^-^ GO OFs (85). High-dose IFN-γ (1000 U/ml) alone was a potent stimulant of MCP-1 in GO and non-GO OFs as well as GO OF-differentiated adipocytes (86, 87) and induced IL-8 secretion with long term incubation (24 hours) (86). However, low- and medium-dose IFN-γ (100-500 U/ml) alone did not up-regulate IL-6 or IL-8 expression in GO and control OFs as well as their corresponding subsets (77, 85, 88), but greatly promoted IL-6 and IL-8 production provoked by CD40-CD40L signaling in mixed GO and control OF populations (77) as well as pure CD90^+^ and CD90^-^ GO OF subsets (18, 85).

Another major pathological role of IFN-γ is establishment of a positive inflammatory feedback loop that maintains Th1 immune response in GO. The serum levels of chemokine C-X-C motif ligand (CXCL) 10 were higher in GO patients than control subjects, especially in active disease (23). Dose-dependent secretion of CXCL9, CXCL10, and CXCL11 after IFN-γ treatment has been observed in GO OFs as well as GO OF-differentiated adipocytes (23, 24). Although TNF-α alone did not induce secretion of the chemokines in GO OFs and adipocytes, IFN-γ further promoted MCP-1, CXCL9, CXCL10, and CXCL11 release stimulated by TNF-α in these cells (23, 24, 87). The proinflammatory effect that IFN-γ with TNF-α synergistically exerts on GO OFs and adipocytes is suppressed in a dose-dependent manner by PPAR-α agonists fenofibrate, gemfibrozil, or ciprofibrate, and PPAR-γ agonists rosiglitazone or pioglitazone (23, 24, 87, 89). A study regarding the role of circulating CXCL9 and CXCL10 as potential markers for GO activity revealed that GC treatment and teleradiotherapy significantly decreased CXCL9 and CXCL10 serum concentrations compared with basal values in GO patients. A positive correlation between CXCL9 and CXCL10 was also found in this study (90). Because C-X-C chemokine receptor (CXCR) 3 is particularly expressed on Th1 cells, which binds CXCL9, CXCL10, and CXCL11 (91), the above studies reflect an accurately self-modulated Th1 immunity-mediated inflammatory network in GO.

Furthermore, IFN-γ results in the accumulation of ECM in GO. IFN-γ enhances hyaluronan synthesis activated by CD40-CD40L signaling in GO OFs and strengthens IL-1β-induced hyaluronan synthesis in GO OFs by promoting expression of the hyaluronan synthase-2 gene (21, 25). It does not directly induce PGE_2_ secretion in GO OFs or contribute to PGE_2_ levels initiated by CD40-CD40L signaling (21). However, IFN-γ acts synergistically with CD40-CD40L signaling to elicit a dramatic increase in PGE_2_ production in CD90^+^ GO OFs and CD90^-^ GO OFs *via* up-regulation of PGSH-2 proteins (85). Conversely, IFN-γ attenuates IL-1β-provoked PGE_2_ production in GO OFs *via* down-regulation of PGHS-2 mediated by decreased *Pghs-2* promoter activity and weakened PGHS-2 mRNA stability. This process is regulated by Janus kinase 2 signaling (25). The different modulation of PGE_2_ production by IFN-γ in combination with other molecular signals indicates a potential role of Th1 cell immunity and its related cytokines in regulating tissue reactivity and remodeling in the orbit. It is recognized that CD90^+^ OFs tend to differentiate into myofibroblasts, a hallmark of late GO fibrosis, whereas CD90^-^ OFs tend to differentiate into adipocytes (2, 6, 22). IFN-γ blocks TGF-β-induced α-smooth muscle actin (SMA) expression in CD90^+^ GO OFs, which inhibits myofibroblast differentiation (22). Similarly, high levels of tissue inhibitor of metalloproteinase (TIMP)-1 gene and protein expression associated with fibrosis have been observed in IL-1β-treated GO OFs in a dose- and time-dependent manner, which was attenuated by IFN-γ *via* down-regulation of *Timp1* promoter activity (26). This suggests that IFN-γ is more of a kind of proinflammatory factor that causes tissue damage and degeneration, and proves that the Th1 immune reaction is predominantly involved in early active GO.

The pathological effects of Th2 cytokines on OFs have yet to be examined carefully (**Figure 3**). Studies in GO murine models have not been able to duplicate Th2-dominated immune responses. A decreased frequency of CD4^+^ IL-4-producing splenic T cells has been observed in hTSHR-A subunit-expressing adenovirus-immunized GO BALB/c mice (36). However, compared with wild type mice, expression of *Il4*, *Il5*, and *Il13* was increased in periorbital tissues of GO SKG mice (48). In another study, serum IL-4 remained at a higher level in hTSHR-A subunit plasmid-immunized GO BALB/c mice than in normal mice with extension of the immune time when IL-6, TNF-α, and granulocyte-macrophage colony stimulating factor were gradually declining (92). These results imply a possible role of Th2 cell-triggered immune responses in orbital connective tissues of stable GO. We used flow cytometry to confirm that the frequencies of CD3^+^CD8^-^ IL-13-producing T cells and CD3^+^CD8^-^GATA3^+^ T cells were augmented in orbital connective tissues from GO patients. Both IL-13 and GATA3 were significantly related to GO development in a multivariate logistic regression model (31). These results suggest an indispensable and major role of Th2 immunity in GO inflammation. Although IL-4 cannot up-regulate CD40 expression in fibroblasts (76), it has many similar effects in regulating the biological behaviors of GO OFs. IL-4 suppresses *Timp1* promoter activation by IL-1β, which reduces the levels of TIMP-1 gene and protein expression in GO OFs (26). IL-4 also suppresses *Pghs-2* promoter activation by IL-1β, thereby inhibiting secretion of PGE_2_ from GO OFs (25). However, IL-4 promotes IL-1β-initiated hyaluronan synthesis in GO OFs by up-regulating hyaluronan synthase-2 gene expression (25). The identical functions of IFN-γ and IL-4 suggest transition from Th1 to Th2 cells to maintain the delicate balance between ECM production and degradation in orbital connective tissues as GO progresses from the early to late stage. In view of the major involvement of Th2 cell immunity in tissue fibrosis (93), more research on the relationship between Th2 cytokines IL-4, IL-5, and IL-13 and GO tissue remodeling is needed.

## Emerging Role of the Th17 Immune Response

The first evidence regarding the possible role of Th17 cells in GO pathogenesis was published in 2008. A total of 216 GD patients and 368 control subjects were genotyped for single nucleotide polymorphisms of *Il23r*. rs2201841 was strongly associated with GO, especially AA (*P*=1.0×10^-4^; OR=2.4) and CC (*P*=1.4×10^-4^; OR=2.36) genotypes (27). This indicates that *Il23r* variants may increase susceptibility to GO by regulating the expression or function of IL-23R on Th17 cells. Soon after, Kim et al. reported significantly higher detectable rates and serum levels of IL-17A in GO patients than those in control subjects, especially in the active phase (94). This was confirmed by another study in which serum IL-17A was higher in both active and inactive GO patients than in control subjects, despite its relative reduction compared with GD patients without eye disease (95). Additionally, Wei et al. observed the highest levels of serum IL-17A in active GO patients compared with those in both inactive GO and GD patients (96). Other studies that focused on lacrimal glands and the ocular surface have revealed elevated IL-17A levels in the tears of active and inactive GO patients (97–99). An orbital magnetic resonance scan showed that the axial lacrimal gland area was positively correlated with IL-17A concentrations in GO patient tears (99). Both serum and tear IL-17A levels have been positively correlated with the GO clinical activity score (94, 96, 99). We also observed up-regulated serum levels of IL-17A, but not IL-17F, in GO patients (44). More importantly, IL-23 (44, 94), IL-6 (44, 95, 97–99), and IL-1β (44, 97–99) concentrations were elevated in both sera and tears from active and inactive GO patients and more enriched in active phase, which are crucial factors for the differentiation of Th17 cells (100, 101). Analogously, the expression of IL-17A, IL-23, IL-6, and IL-1β increases diffusely around small vessels or fibroblasts and adipocytes within GO orbital connective tissues (44). These cytokines may construct a suitable microenvironment for the survival and activation of Th17 cells both systemically and locally in GO. We found that CD3^+^ IL-17A-producing T cells were increased among GO PBMCs compared with controls. Furthermore, both CD3^+^CD8^-^ (Th17) and CD3^+^CD8^+^ (Tc17) IL-17A-producing subsets are augmented in GO peripheral blood (44, 45). The CD3^+^CD8^-^ T cells in GO also express a higher proportion of retinoic acid receptor related orphan receptor (ROR)-γt, the key transcription factor for Th17 cells (44). Intriguingly, most Th17 and Tc17 cells are CD45RO^+^ memory T cells (30, 44, 45), which indicates that these IL-17A-producing T cells might have been exposed to autoantigens such as TSHR and activated in the very early phase of GO or even in the GD stage. This is supported by the fact that the frequency of peripheral Th17 cells is higher in new-onset and intractable GD patients (102–104). More importantly, IL-17A-producing and RORγt-bearing Th17 cells were recruited at a higher fraction in GO orbital connective tissues, which were significantly associated with GO occurrence in a multivariate logistic regression model (31).

Th17 cells facilitate the inflammatory state of OFs in GO autoimmunity (**Figure 3**). In our *in vitro* model, IL-17A promoted transcriptional and translational expression of IL-6, IL-8, and MCP-1 in GO OFs in a dose- and time-dependent manner compared with fibroblasts derived from eyelid tissues. However, IL-17A did not affect the production of IL-23, IL-1β, or TGF-β in GO OFs (44). IL-17A alone did not stimulate RANTES (regulated upon activation, normal T-cell expressed and secreted) production, but strongly induced its mRNA and protein expression in the presence of CD40-CD40L signaling in both GO and control OFs in a dose- and time-dependent manner (45). In a Th17 cell-OF coculture system, Th17 cells promoted the secretion of IL-6, IL-8, MCP-1, macrophage inflammatory protein (MIP)-3, TNF-α, and granulocyte-macrophage colony stimulating factor from both CD90^+^ and CD90^-^ OFs (30). In recent years, circulating fibrocytes have been recognized to participate in GO inflammation and tissue remodeling (105, 106). These cells express CD45, CD34, CXCR4, collagen I, thyroglobulin, TSHR, and IGF-1R, and were far more frequent in the circulation of GD and GO patients than in control subjects and were highly detected in GO orbital connective tissues, but were absent in control orbits (28, 29, 107). Both GO and control fibrocytes secreted TNF-α, IL-6, IL-8, IL-12, MCP-1, RANTES, MIP-1a, MIP-1b, CXCL10, and granulocyte colony-stimulating factor when stimulated by TSH or M22, a monoclonal TSHR-activating antibody (28, 29, 108). We found that GO and control fibrocytes synthesized IL-6, IL-8, and MCP-1 robustly in response to IL-17A, while GO fibrocytes had higher levels of basal and induced secretion of these cytokines than control fibrocytes (32). In a Th17 cell-fibrocyte coculture system, we found that expression of *Il6*, *Il8*, *Mcp1*, *Mip3a*, *Tnfa*, *Cxcl9*, and *Cxcl10* was augmented in GO fibrocytes and their proteins had accumulated in the culture supernatants (32). Both fibrocytes and OFs as well as OF subsets delineated by CD90 express IL-17RA (30, 32, 44), which suggests consecutive stimulation by Th17 cells from peripheral circulation to local orbital connective tissues in GO.

Th17 cells also modulate the fibrosis and adipogenesis balance in GO OFs. IL-17A directly leads to various ECM depositions in orbital connective tissues. Compared with control OFs, the gene and protein synthesis of fibronectin, collagen I, collagen III, TIMP-1, TIMP-2, matrix metalloproteinase (MMP)-1, and MMP-2 was greatly induced by IL-17A treatment of GO OFs in a dose- and time-dependent manner (44). Up-regulation of α-SMA gene and protein expression has been observed in IL-17A-treated GO OFs, which demonstrates differentiation of OFs into myofibroblasts (44). Unexpectedly, when we used pure CD90^+^ and CD90^-^ GO OF subsets, IL-17A exerted distinct effects on the two cell types. Low-dose IL-17A (10 ng/ml) was sufficient to enhance the fibrotic process marked by increased protein levels of α-SMA, fibronectin, collagen I, TIMP-1, and MMP-2 in GO OFs provoked by TGF-β. However, both low- and high (100 ng/ml)-dose IL-17A interfered with adipogenic differentiation of CD90^-^ OFs induced by 15-deoxy-Δ^12,14^-PGJ_2_. The protein levels of perilipin A, adipocyte differentiation-related protein, adiponectin, and PPAR-γ were down-regulated in CD90^-^ OFs in the presence of IL-17A (30). IL-17A promoted phosphorylation of JNK/c-Jun in CD90^+^ OFs, but impeded phosphorylation of CEBP/α in CD90^-^ OFs. Additionally, in CD90^+^ OFs, proteomics analysis has revealed that IL-17A enhances the production of ECM and proteins that are positive regulators for TGF-β and JNK cascade, but prevents adipocyte differentiation of CD90^-^ OFs by up-regulating proteins involved in fatty acid oxidation, degradation, and efflux processes (30). Owing to the considerably high proportion of the CD90^+^ phenotype among GO OFs (30, 109), these findings suggest that GO OFs have a repertoire of differentiation that is more skewed towards myofibroblasts under IL-17A stimulation.

However, GO OFs regulate the phenotype and function of Th17 cells. In a Th17 cell-OF coculture system, both CD90^+^ and CD90^-^ GO OFs enhanced the secretion of IL-17A from Th17 cells. Other supernatant-enriched cytokines included IL-22 and IL-21. An increased frequency of IL-17A^+^RORγt^+^ Th17 cells was shown by flow cytometry in the coculture system, which was repressed by down-regulating PGE_2_ released from CD90^+^ and CD90^-^ GO OFs (30). The molecular mechanisms were possibly mediated by up-regulating IL-23R and IL-1R expression on Th17 cells, which was caused by PGE_2_-EP2/EP4 signaling that led to intracellular cAMP formation and subsequent phosphorylation of cAMP-responsive element-binding protein (31). These *in vitro* findings are consistent with the observation that GO orbital connective tissues contain a level of PGE_2_ and orbit-infiltrating Th17 cells express more IL-23R and IL-1R (31). Moreover, the Th17 cell-OF interaction results in a dramatic elevation of the expression of CD40, MHC II, ICAM-1, and VCAM-1 on CD90^+^ and CD90^-^ GO OFs, particularly on those that are also CD34^+^ (30). Such CD34^+^ OFs may originate putatively from CD34^+^ fibrocyte progenitors (106). Flow cytometric analysis has shown that CD34^+^ GO OFs have higher levels of IL-17RA than native residential CD34^-^ subsets, which might account for the overexpressed CD40 and MHC II on CD34^+^ cells (31). Moreover, Th17 cell-fibrocyte interplay not only enhances IL-17A production in Th17 cells, but also significantly promotes CD40 and MHC II expression on GO fibrocytes (32).

How are Th17 cells recruited into orbital connective tissues in GO? Both peripheral and orbit-infiltrating Th17 cells express C-C chemokine receptor (CCR) 6, a MIP-3 receptor (30–32). Therefore, the MIP-3 released by GO fibrocytes might be a strong attractant that directs Th17 cells to sites of inflamed orbital connective tissues. Guo et al. demonstrated that orbit-infiltrating T cells in GO express CD44 (110), a specific cell surface receptor for hyaluronan (111). CD44 is highly elevated on activated T cells (112, 113) and particularly on CCR6^+^ IL-17A-producing Th17 cells in our study (30). However, T cell subsets with low expression of CD44 hardly secrete IL-17A in GO patients (30). Thus, with increased pericellular hyaluronan deposition, CD44 may facilitate Th17 cell attachment to GO OFs.

In recent years, the concept of Th17 cell plasticity has become prominent. Th17 cells acquire much more complex functional phenotypes than previously thought. Although they can shift phenotype within their lineage, Th17 cells have a dynamic ability to trans-differentiate into other CD4^+^ T cell subsets such as Th1 and Th2 cells (100, 114, 115). IFN-γ- and IL-22-producing Th17 cells are detected at significantly higher levels among GO PBMCs, especially in active patients (30, 45). These so called pathogenic Th17 cells express both RORγt and Tbet. They infiltrate into GO orbital connective tissues and more likely produce IFN-γ instead of IL-17A (31). TSH and M22 robustly induced gene and protein expression of IL-23 in GO fibrocytes, but not IL-12, which was significantly produced by GO OFs under the same conditions (34). However, pure CD34^+^ OFs preferentially expressed *Il23p19*, while their homologous CD34^-^ OFs greatly expressed *Il12p35 (* 34). The distinct roles of CD34^+^ and CD34^-^ OFs reflect the potential shift from a non-pathogenic to pathogenic state of circulating Th17 cells into orbit-infiltrating Th17 cells, which is consistent with the TSHR signaling that drives the specific cytokine milieu by CD34^+^ fibrocytes that masquerade as CD34^+^ OFs within orbital connective tissues. The expression of IL-23 by CD34^+^ fibrocyte/OF lineages might play a prominent part in reinforcing the highly IL-23R-bearing Th17 phenotype in GO orbits (31) by endowing Th17 cells with “pathogenic” effector functions. We recently reported an increase in peripheral classic CD3^+^CD8^-^CXCR3^+^CCR6^-^ Th1 cells in active moderate-to-severe GO patients and GD patients, which were decreased in active very severe GO patients. Conversely, we found that classic CD3^+^CD8^-^CXCR3^-^CCR6^+^ Th17 and non-classic CD3^+^CD8^-^CXCR3^+^CCR6^+^ Th17.1 cells were elevated among PBMCs from active very severe GO patients compared with both active moderate-to-severe GO and GD patients. Intriguingly, the non-classic Th17.1 cells favored IFN-γ production in active very severe GO patients, but preferentially secreted IL-17A in active moderate-to-severe GO patients. Moreover, the peripheral Th17.1 cells expressed higher levels of RORγt in active moderate-to-severe GO patients, whereas they had augmented levels of Tbet in active very severe GO patients, which was in concert with the different cytokine production phenotypes of these two patient cohorts. Very severe GO patients who did not respond to intravenous GC treatment had a sustained higher frequency of circulating and orbit-infiltrating Th17.1 cells (33). Therefore, we speculate an immunological transition process from Th1 cell immunity to Th17 cell immunity may indicate the development of very severe eye disease in GD. The overactivity of Th17.1 cells may serve as a hallmark for the not yet subsided inflammatory storm in orbital connective tissues. Evidence from animal models is indicating that IL-17A and IFN-γ double-producing Th17 cells are pathogenic drivers of various human autoimmune diseases such as multiple sclerosis, diabetes type 1, uveitis, dry eye, rheumatoid arthritis, and inflammatory bowel disease (100, 114). Unfortunately, no convincing evidence of detectable Th17 cells has been observed in current GO murine models (36, 53), which makes it difficult to prove our hypothesis. The distinctive genetic backgrounds of BALB/c and C57BL/6J mice may partially be responsible for their susceptibility to GD and GO as well as the different T cell responses under autoimmune disease conditions (116). In this regard, a role of the gut microbiota that influence the immunological responses of induced GO murine models cannot be neglected (37, 117). For example, the YCH46 strain of *Bacteroides fragilis* reduces Th17 cell numbers by releasing propionic acid in GD patients (118). An interesting study reported correlations between murine GO manifestation and gut microbial taxonomies. Significant differences in the diversity and spatial organization of the gut microbiota of hTSHR-A subunit plasmid-immunized BALB/c mice were shown in two centers from different countries (37). Thus, the impact of different regions is also a source of potentially conflicting results, since the microbiome changes across different countries. Disease-associated gut microbiota may contribute to the induced immune responses in GO murine models. Despite the confounding deviation from real human GO, future animal models will certainly be developed from existing experience and provide researchers with novel points of study to investigate the immunopathogenesis of GO.

## Future Perspectives

To date, immunomodulation therapy has been widely used for treatment of GO. Traditional non-specific immunosuppressants are effective in combination with GC treatment as alternative options for active moderate-to-severe GO (8, 11). Azathioprine and methotrexate interfere with purine synthesis that is necessary for lymphocyte proliferation. Mycophenolate, which inhibits inosine monophosphate dehydrogenase, and cyclosporine, which prevents IL-2 secretion, also exert anti-proliferative effect on lymphocytes (8, 11). However, none of these therapeutic approaches appear to alter the natural course of GO, which makes development of more specific drugs critical to address an important unmet medical need. Considering the complexity of GO pathogenesis, there remain many ambiguous aspects of the pathological T cell activities within orbital connective tissues. For example, T cell migration and activation induced by autoantigens, autoantibodies, and immunomodulatory proteins. Activating TSHR on thymocytes enhances thymic output and therefore the functional T cell repertoire in the periphery (119). A larger proportion of peripheral CD3^+^CD45RO^+^IGF-1R^+^ T cells is seen in GO patients compared with control subjects. IGF-1R, which increases upon TCR stimulation, not only inhibits Fas-mediated apoptosis, but also supports the expansion of memory T cells in GO (120). Furthermore, the proportion of peripheral IGF-1R^+^ T cells declines with clinical improvement in GO patients after rituximab treatment (121). Autoantibodies from GO patients up-regulate T cell chemoattractant IL-16 and RANTES from GO OFs (122). Moreover, T cell immunoglobulin domain and mucin domain 3, which restrains cytokine production in effector T cells except Th2 cells, is down-regulated in peripheral Th1 and Th17 cells in GO patients (123, 124). Slit2 from residential CD34^-^ OFs might inhibit production of IL-6 from GO CD34^+^ OFs, thereby ameliorating orbital inflammation and repressing Th17 cell differentiation (125). These findings offer new insights to explore novel approaches for therapy of GO. Existing evidence for the efficacy and relative safety of rituximab against CD20^+^ B cells, tocilizumab against IL-6, etanercept, infliximab, and adalimumab against TNF-α is encouraging (7, 71, 126). The impressive results of teprotumumab have provided the unprecedented possibility for monoclonal antibodies in combination with GCs for GO therapy, although more evidence must be provided. Trials of utilizing belimumab against BAFF (EUDRACT 2015−002127−26), K1-70 against TSHR (NCT02904330), and iscalimab against CD40 (NCT02713256) are currently underway. Blocking the IL-23/IL-17A axis as a therapeutic strategy for GO is also promising considering its effectiveness in other autoimmune diseases such as psoriasis and mandatory spondylitis (71). Notably, a recent interesting study had the first attempt of antigen-specific immunotherapy with ATX-GD-59 that contains two TSHR peptides 9B-N and 5D-K1 in GD, which suggests that ATX-GD-59 is a safe and well-tolerated treatment (127). This antigen-specific method blocks the activation of APCs by binding with HLA-DR molecules, thereby inhibiting the subsequent cascade reactions of self-reactive T and B cells, which truly represents the needed breakthrough for targeted and effective therapy with less prone to general side effects. Novel biological agent identification on the basis of advances in GO pathogenesis is time-consuming but rewarding, which ultimately benefits patients with this debilitating disease.

## Author Contributions

SF and YH wrote the paper. YL constructed all the figures. HZ revised the paper. XF was responsible for the writing idea and framework of the paper. All authors contributed to the article and approved the submitted version.

## Funding

This work was supported by the National Natural Science Foundation of China (81930024, 81761168037, 81770974, 81800695, 82071003, 82000879, 81570883, 81600766, 31701046, 31600971, and 31500714), the National Key R&D Program of China (2018YFC1106100, 2018YFC1106101), the Shanghai Sailing Program (18YF1412300), the Research Grant of the Shanghai Science and Technology Committee (20DZ2270800, 17DZ2260100, 19410761100, and 19DZ2331400), the Clinical Research Plan of SHDC (SHDC2020CR3051B), the Project of Medical Robots (IMR-NPH202002) From the Clinical Joint Research Center of the Institute of Medical Robots, Shanghai JiaoTong University-Shanghai Ninth People’s Hospital, the Collaborative Research Project of Translational Medicine Collaborative Innovation Center, Shanghai JiaoTong University School of Medicine (TM201718), the Shanghai Municipal Education Commission—Gaofeng Clinical Medicine Grant Support (20152228), the Shanghai JiaoTong University Translational Medicine Crossed Research Grant (ZH2018ZDA12, ZH2018QNA07), the Sample Database Project of Shanghai Ninth People’s Hospital (YBKB201901), and the Joint Innovation Team for Young Physicians of Shanghai Ninth People’s Hospital (QC202002).

## Conflict of Interest

The authors declare that the research was conducted in the absence of any commercial or financial relationships that could be construed as a potential conflict of interest.
